# *In vitro* inhibition of voltage-dependent sodium currents by the antifungal drug amorolfine

**DOI:** 10.1016/j.jbc.2025.108407

**Published:** 2025-03-14

**Authors:** Mohammad-Reza Ghovanloo, Sidharth Tyagi, Philip R. Effraim, Stephen G. Waxman

**Affiliations:** 1 Department of Neurology, Yale School of Medicine, New Haven, Connecticut, USA; 2 Center for Neuroscience & Regeneration Research, Yale University, West Haven, Connecticut, USA; 3 Neuro-Rehabilitation Research Center, Veterans Affairs Connecticut Healthcare System, West Haven, Connecticut, USA; 4 Medical Scientist Training Program, Yale School of Medicine, New Haven, Connecticut, USA; 5 Department of Anesthesiology, Yale School of Medicine, New Haven, Connecticut, USA

**Keywords:** amorolfine, voltage-gated sodium channel, patch-clamp, pharmacology, voltage clamp, physicochemical properties, modulated receptor hypothesis, ultra-hydrophobic pathway, Nav1.7

## Abstract

Voltage-gated sodium (Nav) channels are critical for electrical signaling, and their pharmacological modulation can be leveraged for the development of therapeutic agents targeting various disorders. The local anesthetic (LA) site on Nav channels is particularly important, as it is a common target for many clinically used inhibitors, including anticonvulsants and antiarrhythmics. Our goal was to identify novel Nav channel inhibitors by leveraging physicochemical criteria, focusing on potential LA site binding candidates. We identified amorolfine (AMF), a phenyl-propyl morpholine derivative, as a putative modulator. Our results demonstrate that AMF acts as a state-dependent inhibitor of Nav channels, with a ∼30-fold preference for inactivated states. It stabilizes channel inactivation and prevents channel from conducting, driven through its stabilization of inactivation. These findings suggest that AMF represents a new compound that inhibits Nav channels, offering insights into the development of future therapeutic agents targeting Nav and potentially other ion channels.

The sodium current passing through voltage-gated sodium (Nav) channels initiates and propagates action potentials in various excitable cells (1). Nav channels are hetero-multimeric proteins comprised of large α-subunits and smaller auxiliary β-subunits (2, 3). The α-subunit (of which there are nine isoforms, Nav1.1–9) is expressed by a single transcript composed of four homologous six-transmembrane α-helical segment domains, known as DI-DIV. Each of these structural domains is functionally divided into a voltage-sensing domain (VSD) and a pore-domain (PD). These functional domains are linked through an S4-S5 linker peptide that controls the channel gating (opening and closing) in response to voltage changes (2, 4, 5, 6, 7).

During depolarization, the Nav channels open as the positively charged S4 segments in the VSDs move outward, pulling on the S4-S5 linkers, and opening the PD. Within milliseconds, Nav channels undergo fast inactivation, mediated *via* an allosteric interaction of three key residues in the DIII-DIV linker (IFM motif) with a restriction ring on the channel's intracellular side (8, 9, 10, 11). Additionally, Nav channels can enter slower inactivated states due to repetitive or prolonged stimulations (12).

From a pharmacological perspective, targeting each of these structural components of the Nav channels can modulate their physiological function, providing avenues for treating various disorders. The most well-known pharmacological binding site is the local anesthetic (LA) site (*e.g.*, lidocaine), located on the intracellular side of the PD (4, 10, 13, 14, 15, 16). This site is accessible through the activation gate (V-gate) and through the intralipid openings around this site, known as channel fenestrations (17, 18, 19). Blocking schemes for both charged and neutral compounds *via* these pathways have been extensively verified since the 1970s (4, 19, 20, 21, 22). In recent years, studies with cannabidiol (CBD) have shown that this ultra-hydrophobic compound (high calculated-LogD of 6.32 [where cLogD is a measure of a compound's lipophilicity that accounts for its ionization state]) interacts with the LA site, as well as a secondary site next to the IFM motif (18, 23, 24).

We previously showed that CBD does not alter open-state fast inactivation in Nav channels, and its resting-state block of Nav1.4 is abolished in mutant channels with fully and partially occluded fenestrations (18, 25). However, other well-known LA compounds, such as lidocaine and flecainide, continued to block the mutant channels in the resting state at equilibrium. This suggested that CBD accesses the LA site through the fenestrations rather than the V-gate. The molecular structure of CBD includes two oxygen atoms on both sides of a benzene ring, with a hydrocarbon tail on one end and a hydrocarbon ring on the other (26). These features create localized electronegativity clusters surrounded by carbon atoms. In contrast, less hydrophobic LA site-binding molecules like lidocaine (cLogD of 2.33) and flecainide (cLogD of 1.01) have a more even distribution of electronegative atoms relative to carbon atoms.

In this study, we hypothesized that physicochemical descriptors, such as partitioning coefficients, molecular weight, and general shape/atom distribution, could be used to identify a compound with similar inhibitory effects to CBD on Nav channels. Therefore, we aimed to find a clinically viable compound fitting this description, not previously linked with ion channel targeting, and test its effects. Through our search of clinically approved drugs, we identified amorolfine (AMF, cLogD of 4.49), a morpholine antifungal drug known to inhibit the fungal sterol synthesis pathway (27, 28). Our results indicate that AMF is a state-dependent Nav channel inhibitor, as tested on the well-defined pain threshold channel Nav1.7 (29), and falls in the blocking scheme category of CBD for a neutral, high LogD drug, thereby suggesting the utility of general physicochemical descriptors for predictive experimental validation.

## Results

### AMF is a state-dependent Nav channel inhibitor

Previous studies have demonstrated that compound size plays a crucial role in interactions that occur at the LA site *via* Nav channel fenestrations (17). Additionally, our prior work with CBD and related compounds has shown that hydrophobicity is another key factor in determining how a compound travels through intralipid fenestrations to reach the LA site (18).

Molecular dynamics simulations further revealed that the atomic distribution within CBD could be a determinant of its localization within the biomembrane leaflets (18). Specifically, CBD's electronegative oxygen atoms cluster on one end, while hydrocarbon-rich regions dominate the other ends. This asymmetry causes CBD to localize just below the membrane's phosphate groups, as shown by molecular dynamics simulations and further verified by nuclear magnetic resonance (NMR) experiments. The oxygen atoms likely prevent CBD from diffusing across membrane leaflets, while its hydrophobic tail keeps it from interacting with external water molecules (18).

Building on these findings, we sought to identify a suitable candidate to test our hypothesis. Using the PubChem, DrugBank, and ChEMBL databases, we screened compounds based on 1) high cLogD, which represents the compound distribution partitioning coefficient (CBD: 6.32), 2) electronegative atom-to-carbon clustering distribution across the molecule, 3) molecular weight (CBD: ∼315 g/mol), and 4) clinical approval for a disorder unrelated to Nav channels with no prior studies on Nav channel activity. Through this search, we identified AMF as a suitable candidate. AMF has a molecular weight of ∼318 g/mol, a cLogD of 4.49, indicating high hydrophobicity, local clustering of two electronegative atoms on one end, and hydrocarbon-rich ring structures on the other. Importantly, no prior studies have implicated AMF in Nav channel activity.

**Figure 1:**
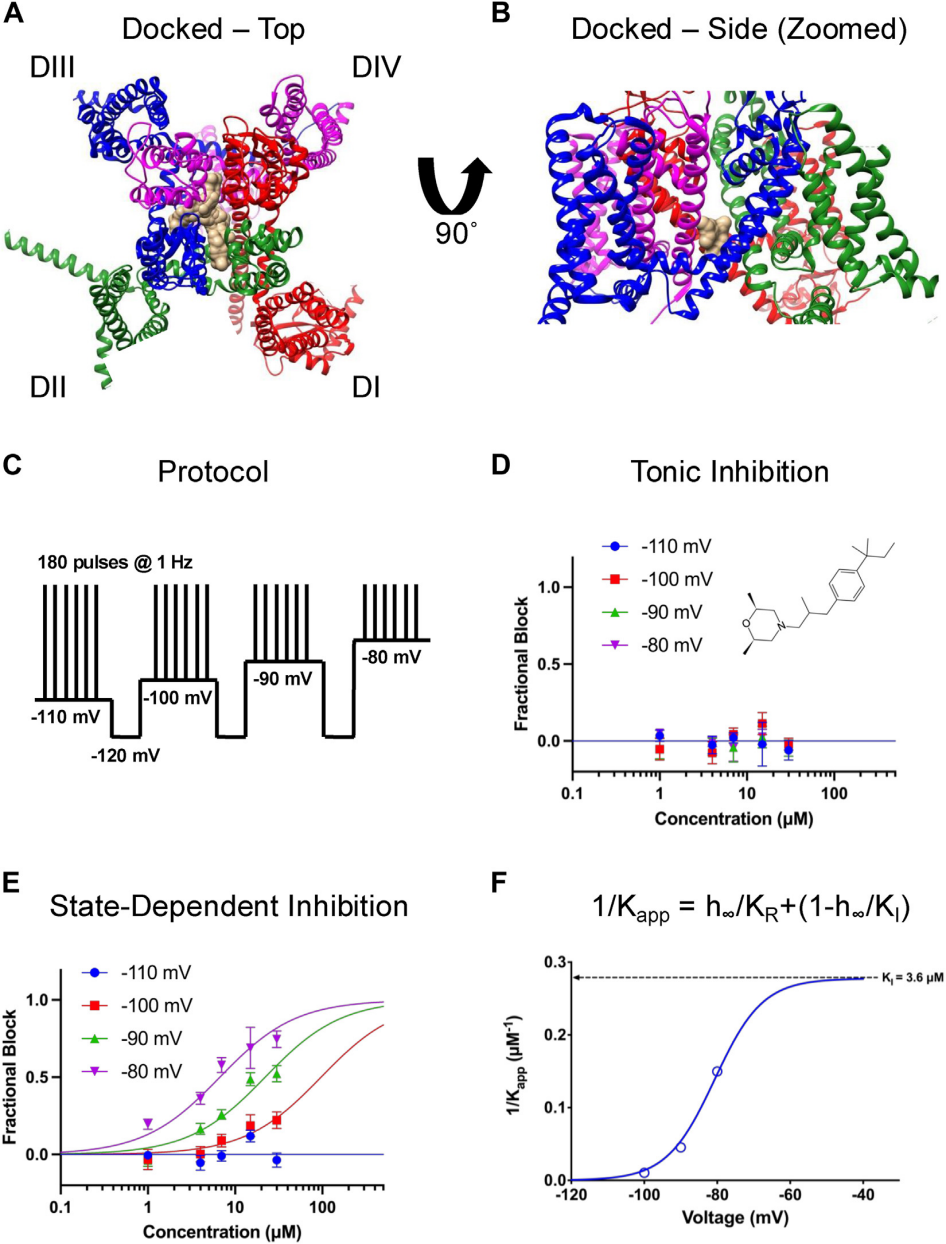
**AMF is a Nav Channel Inhibitor.***A*, top view of AMF docked into the Nav1.7 channel structure. AMF is shown in beige color. *B*, side view highlighting the most favorable binding pose within the LA site. *C*, the protocol used for the inhibition experiments. *D*, Nav current inhibition observed during the first pulse at varying holding potentials (n = 7–15). *E*, inhibition observed at the final holding-potential interval (n = 7–15). *F,* data fitted to a four-state model based on IC_50_ curves from panel E. Data are presented as mean ± SEM.

As noted above, the local clustering of a nitrogen and an oxygen atom on one end of AMF, combined with a series of carbon atoms on the other end of the molecule, gives AMF a shape loosely reminiscent of a CBD molecule (although CBD has no nitrogen atoms) (30). Therefore, we hypothesized that AMF would also interact at the LA site. To test this hypothesis computationally, we performed a molecular docking simulation of AMF with Nav1.7 (Fig. 1, *A* and *B*; Table 1). Our results indicated that all the most favorable binding poses of AMF fall within the LA site and three of the four fenestrations (except the DI/DIV side). These results generally supported the hypothesis that AMF inhibits the channel, thus meriting further experimental investigations.

**Table 1:** Energy associated with the binding of AMF into the pore of Nav1.7 of the top three best positions

Model	Calculated affinity (kcal/mol)
1	−8.628
2	−8.599
3	−8.486

A key hallmark of LA-binding Nav channel inhibitors is an increased apparent potency of inhibition as the channel enters more inactivated states (25, 31). This is because with each depolarization episode, the three-dimensional structure of the LA site adapts a more favorable conformation for ligand binding (17, 31). Therefore, our first experimental objective was to determine if AMF is a state-dependent inhibitor. We used a protocol in which holding potentials varied from −110 to −80 mV, with a recovery pulse to −120 mV between each interval to reset channels to full availability (32). During each interval, we pulsed the channels 180 times at 1 Hz to allow AMF to reach equilibrium with the channels (Fig. 1*C*). We then plotted the first and last pulses from each interval as a function of AMF concentration to construct concentration-response curves. Each cell was exposed to a single concentration of AMF, and the final normalized relationships were pooled and fit with the Hill-Langmuir equation.

Our results indicated that AMF does not tonically inhibit the Nav current at the first pulse from any of the holding-potential intervals (Fig. 1*D*). However, after reaching equilibrium over the course of 180 pulses, AMF state-dependently inhibits the channels, with IC_50_ values ranging from 6.7 to 96.5 μM at holding-potentials of −80 to −100 mV, but without discernible inhibition at −110 mV (Fig. 1*E*). The Hill-slopes for the more depolarized holding-potentials of −90 and −80 mV were ∼0.8 to 1, thus we fixed the slope for −100 mV to 1 to ensure a better fit. This Hill-slope of 1 suggests that there is a singular 1:1 interaction between AMF and Nav channels culminating in inhibition.

In Figure 1*F*, we show a plot of the inverse of the apparent IC_50_, fit with a four-state binding model that used parameters obtained from the Boltzmann fit of the voltage-dependence of steady-state inactivation (SSI) (32). The potency numbers were based on the results shown in Figure 1*E*. This established that the apparent potency is directly related to the proportion of inactivated channels at different holding-potentials. These results demonstrate that AMF inhibits the Nav current with a K_I_ of 3.6 μM (Fig. 1*F*).

### AMF–kinetics of inhibition

**Figure 2:**
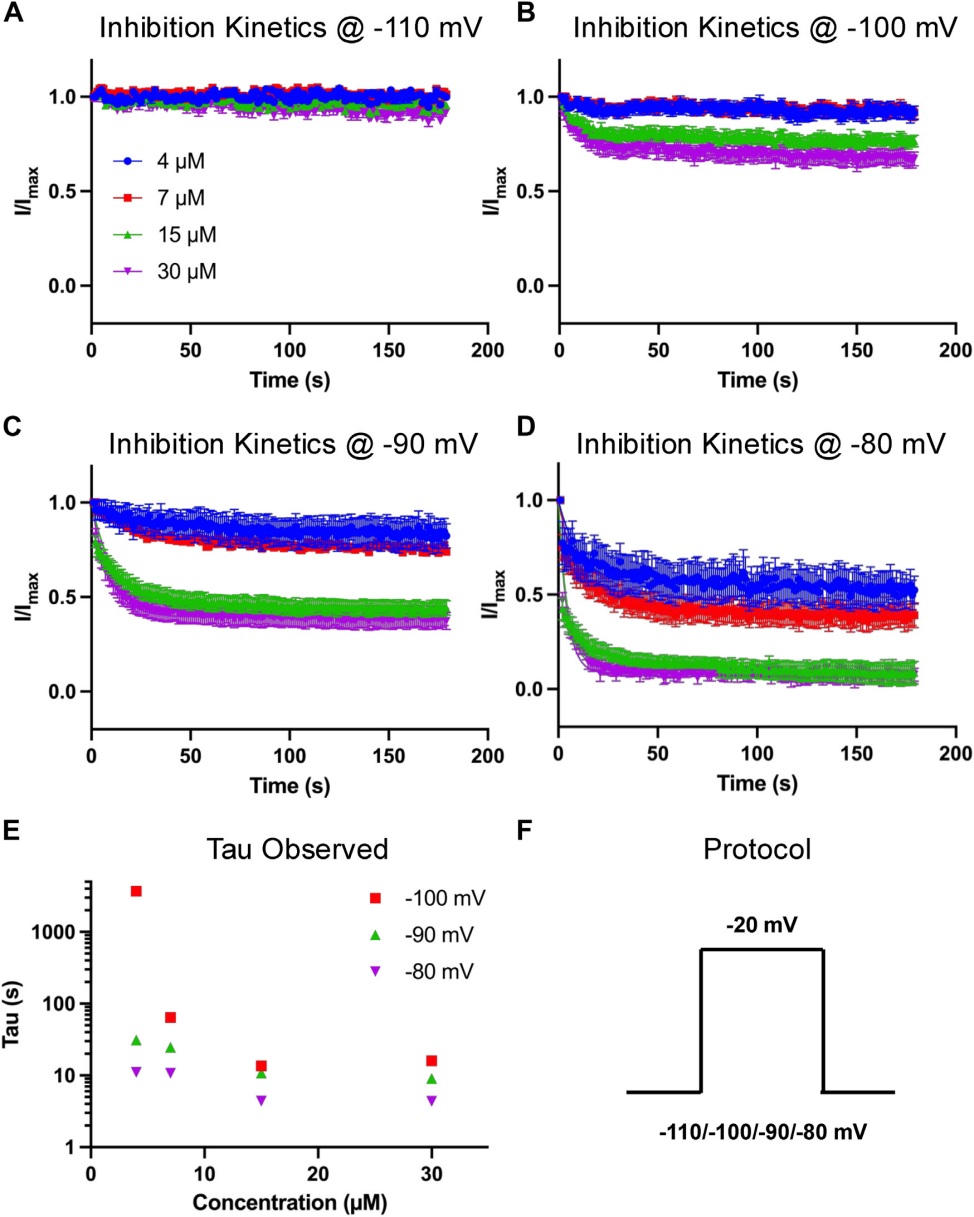
**Kinetics of AMF-Mediated Inhibition.***A-D*, kinetics of inhibition at 4, 7, 15, and 30 μM AMF from the indicated holding-potentials, normalized to vehicle control (n = 5–12). *E*, observed time constants (Tau) determined by fitting the mean data from panels A-D with an exponential function. *F*, the square pulse protocol used to assess inhibition kinetics. Data are presented as mean ± SEM.

Next, we sought to investigate the kinetics of inhibition of AMF on Nav currents. To do this, we measured peak I_Na_ amplitude over 3 min during a series of pulses to −20 mV from the same holding-potentials as before. The observed rates of compound equilibration (as time constant, Tau_Observed_) were determined by fitting a single exponential equation to current decays (Fig. 2, *A*–*F*). We normalized the inhibition fractions across concentrations and at each holding-potential to the response in the vehicle and plotted it *versus* time elapsed after the application of AMF.

Consistent with the IC_50_ curves, AMF inhibited the Nav current faster as the holding potential was depolarized, in a concentration-dependent manner, with the rates at each holding potential increasing. The exception was at −110 mV, where AMF did not induce discernible inhibition of the Nav current (Fig. 2, *A*–*F*). Altogether, these data show that AMF is a state-dependent inhibitor of Nav currents.

### AMF prevents the channel from conducting, but does not alter the voltage dependence of activation

**Figure 3:**
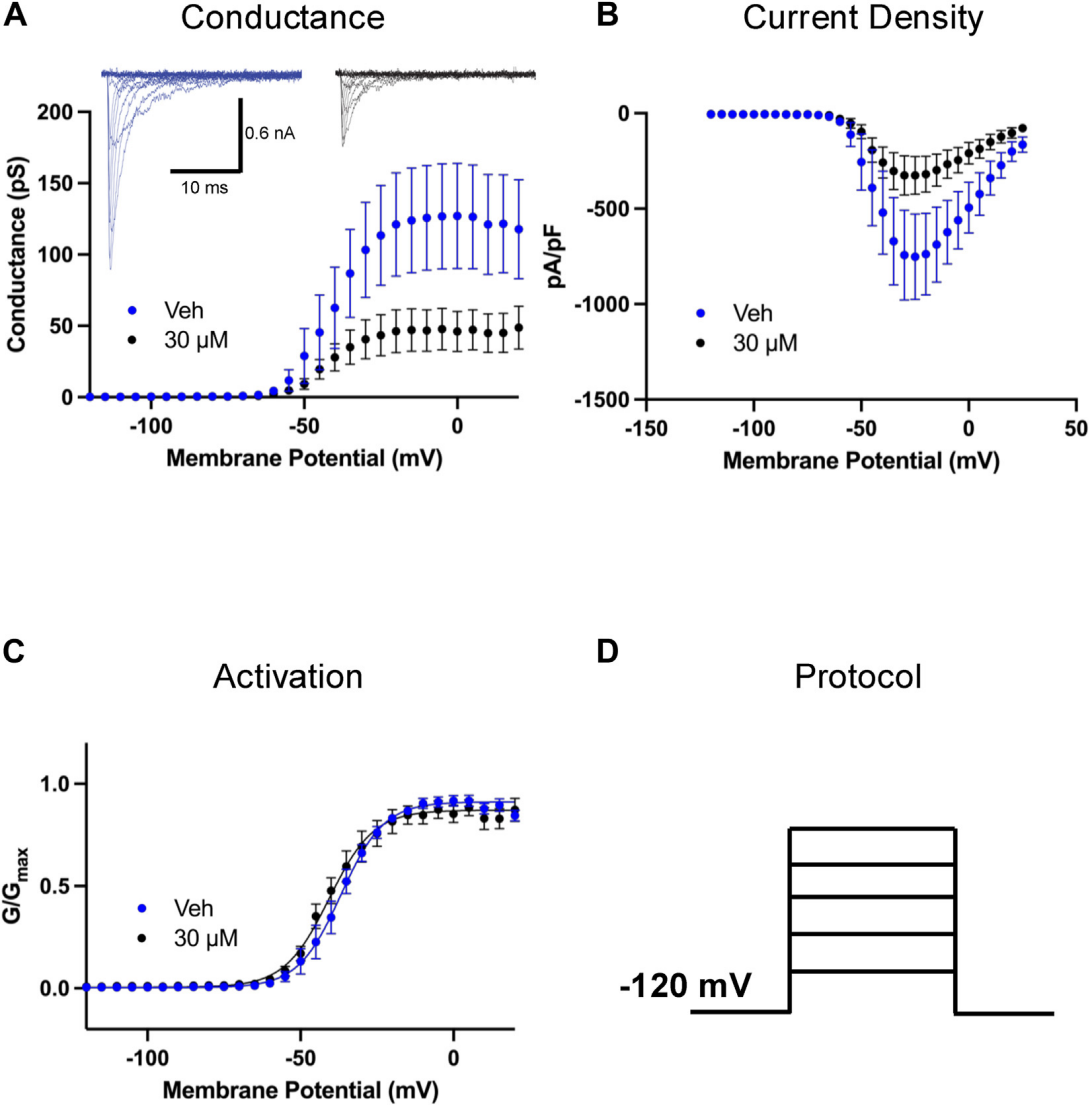
**AMF Prevents Channel Opening Without Altering Activation Voltage-Dependence.***A*, peak conductance measurements of Nav channels (n = 7–8) (*p* < 0.05). Representative families of macroscopic currents are shown as inset. *B*, current density measurements (n = 7–8) (*p* < 0.05). *C*, normalized conductance to assess voltage-dependence of activation (n = 7–8) (*p* > 0.05). *D*, protocol used in the experiments. Data are presented as mean ± SEM.

We then investigated the effects of AMF on Nav activation by measuring peak channel conductance across membrane potentials ranging from −120 to +25 mV. This was done using a series of 500-ms steps, where the peak measured from each step reflects a combination of maximal conductance and inactivation (as inactivation accumulates over 500 ms), thereby representing the apparent maximal conductance at each step (Fig. 3). We observed the impact of 30 μM AMF on peak conductance as a function of membrane potential (Fig. 3*A*). Approximately 50% of sodium conductance was inhibited at this concentration of AMF (*p* < 0.05). In Figure 3*B*, we present a plot of sodium current density, expressed as peak I_Na_ divided by membrane capacitance (pA/pF), as a function of membrane potential. Consistent with the conductance data, there is a ∼50% reduction in magnitude at 30 μM AMF (*p* < 0.05).

The normalized conductance is plotted against membrane potential (Fig. 3*C*), showing that AMF does not shift the midpoint (V_1/2_) of activation of the remaining available fraction of Nav channels (*p* > 0.05). Thus, while AMF exposure at 30 μM prevented about 50% total Nav channels from conducting, it did not alter the voltage-dependence of activation in the other 50% of the channels as measured from holding-potential of −120 mV (Fig. 3*D*). This effect is similar to other ultra-hydrophobic compounds that we have tested in previous studies (18, 25, 33, 34, 35).

### AMF does not alter open-state fast inactivation (FI); AMF hyperpolarizes the steady-state inactivation curves

**Figure 4:**
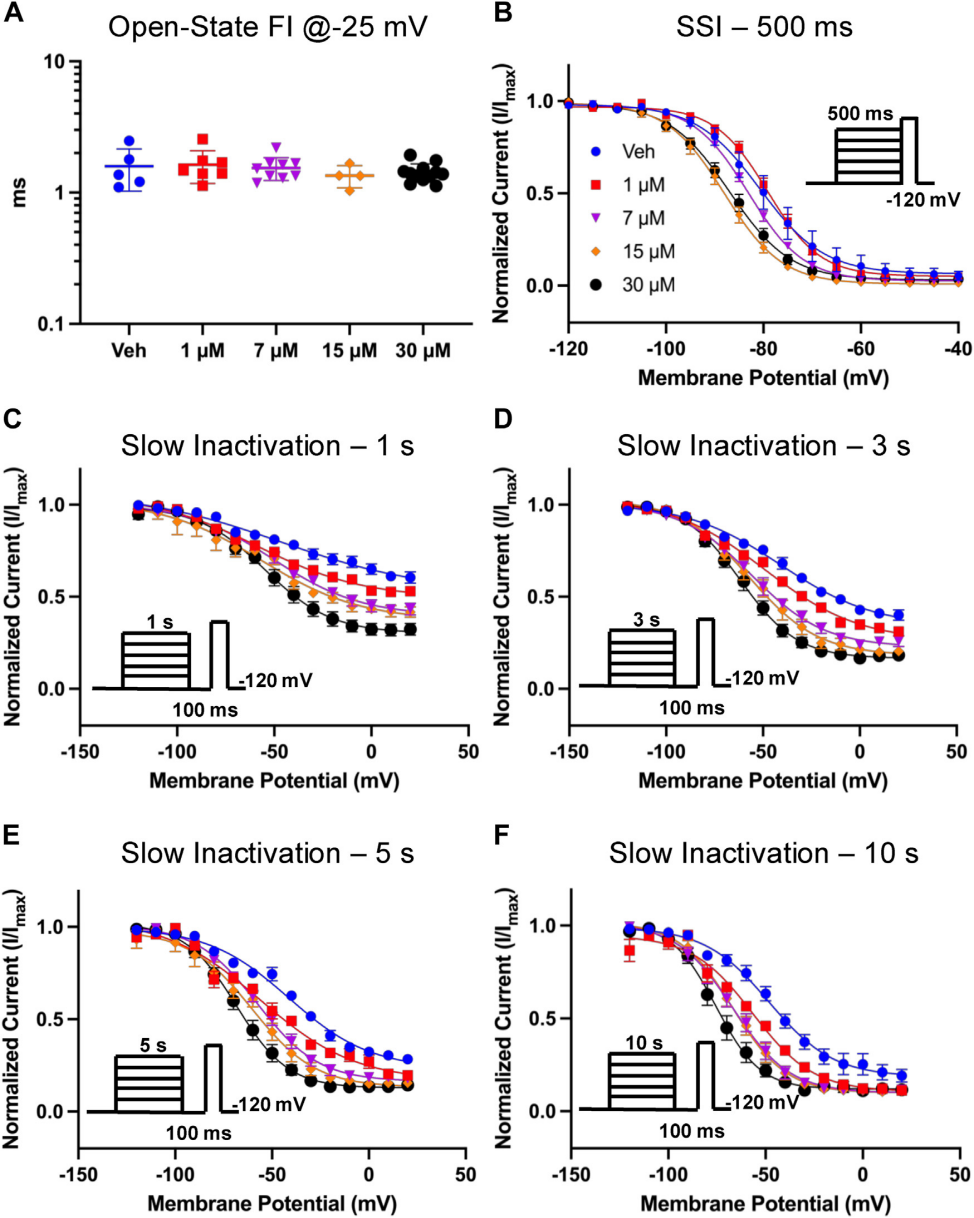
**AMF Effects on Inactivation.***A*, time constants of open-state fast inactivation, measured using an exponential function. The distributive scatter plot is shown as mean ± SD (n = 4–12) (*p* > 0.05). *B,* steady-state intermediate inactivation data (n = 5–13). *C–F,* steady-state slow inactivation measured after 1, 3, 5, and 10 s (n = 6–11). All data are fitted with a Boltzmann function. Data are presented as mean ± SEM.

Our next goal was to determine whether AMF affects open-state FI, or true fast inactivation (36, 37). We measured the time constant associated with open-state inactivation at −25 mV, which was the potential that elicited the maximal peak I_Na_. This was done by fitting an exponential function to the inactivating traces at −25 mV. We found that there were no differences at any of the AMF concentrations compared to vehicle (Fig. 4*A*) (*p* > 0.05). This indicates that AMF does not interact with the open-state of the Nav channel, which is similar to what we have reported with other highly hydrophobic compounds including CBD, cannabigerol (CBG), and cannabinol (CBN) (25, 33, 34, 35, 38). In contrast, traditional LA compounds like lidocaine have been shown to modulate the open-state fast inactivation kinetics of Nav currents in a concentration-dependent manner (39).

To further investigate AMF's effect on inactivation, we measured the voltage dependence of SSI from a pre-pulse duration of 500 ms, which is considered to trigger fast to intermediate inactivation (35, 40). In Figure 4*B*, we show normalized current amplitudes at the test-pulse as a function of pre-pulse voltages. Our results show that AMF hyperpolarizes the SSI curve in a concentration-dependent manner of the remaining fraction of Nav channels that were not prevented from conducting. This indicates that AMF increases the likelihood of the Nav channels that are available to open to inactivate over the course of the pre-pulse duration. The overall effect is inhibition.

The observed hyperpolarization of SSI prompted us to investigate AMF's effects on the Nav slow inactivation at 1, 3, 5, and 10 s (Fig. 4, *C*–*F*). In these experiments, we first held the channels at −120 mV, followed by a series of depolarizing steps for one of the noted durations, which was followed by a hyperpolarizing step back to −120 to recover the fraction of the channels that had entered fast inactivation for 100 ms. Finally, the current amplitude was measured by a test-pulse to −20 mV. We found that AMF also hyperpolarizes the steady-state slow inactivation curves at all time courses in a concentration-dependent manner. Altogether, these results indicate that AMF does not interact with the open-state of the Nav channel but targets the inactivated states of the channel.

### AMF slows recovery from inactivation

**Figure 5:**
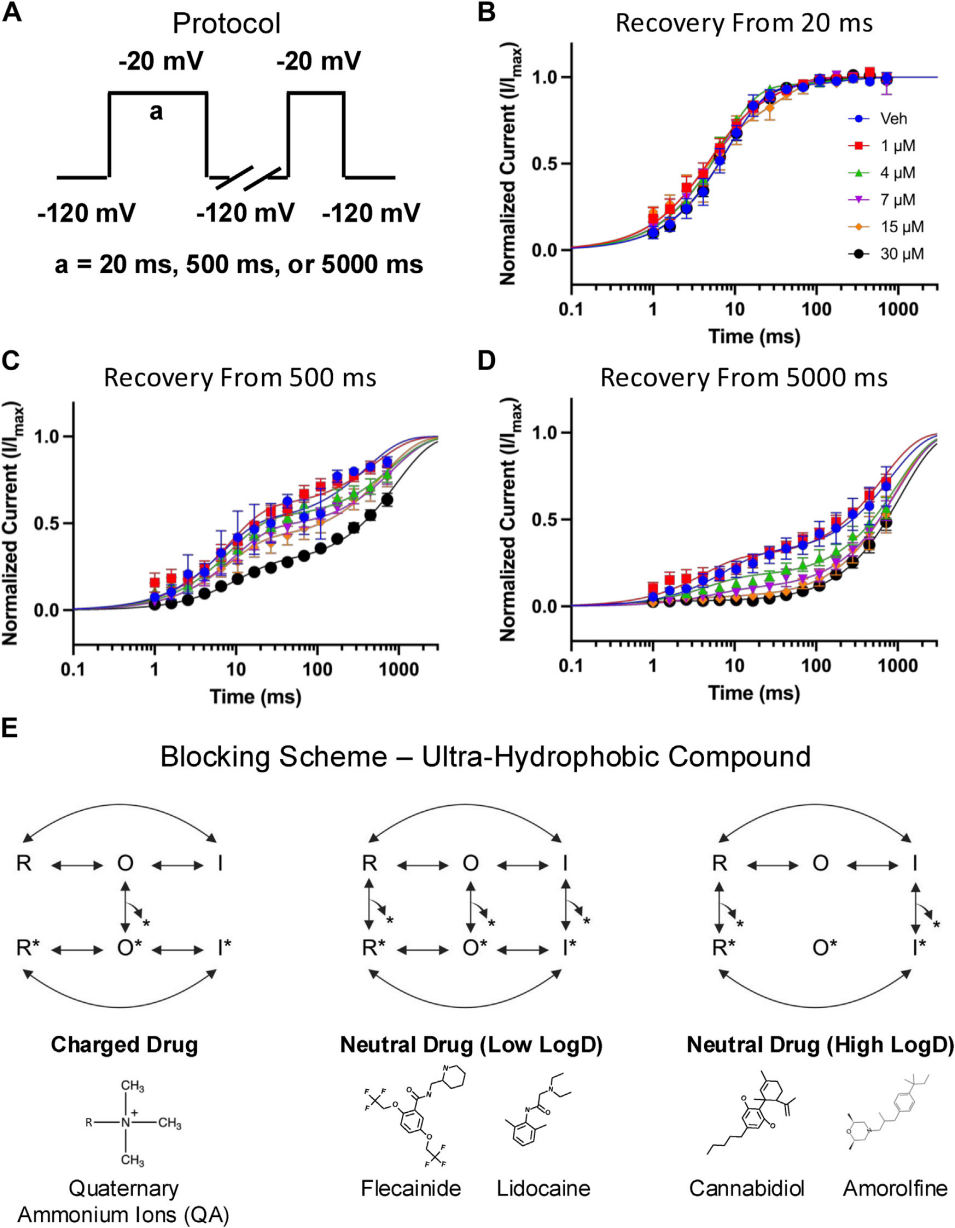
**AMF Effects on Inactivation Recovery and Proposed Blocking Mechanism.***A*, protocol used for inactivation recovery experiments. *B–D*, recovery from inactivation was measured at 20 ms (fast), 500 ms (intermediate), and 5000 ms (slow) intervals, fitted with a bi-exponential function (n = 3–15). *E*, proposed channel blocking scheme (based on modulated receptor hypothesis (4, 19, 20, 21, 22)) for an ultra-hydrophobic compound, illustrating the interaction of charged (hydrophilic) and neutral (hydrophobic) drugs with Nav channel states. The third model is based on our results with CBD, AMF, and Nav channels, highlighting that as a drug becomes more hydrophobic, its interaction shifts from predominantly open-state (O) to rest (R) and inactivated (I) states. The star indicates the drug. Data are presented as mean ± SEM.

To evaluate the time-dependence and extent of AMF's effects on inactivation stabilization, we examined the recovery of Nav channels from fast (20 ms), intermediate (500 ms), and slow (5000 ms) inactivation. Channels were held at −120 mV to ensure full availability, then subjected to a depolarizing pulse to −20 mV for one of the specified durations. Recovery was assessed by measuring the time it took for channels to recover at −120 mV (Fig. 5*A*). The mean normalized currents were plotted and fit with a bi-exponential function, as shown in Figure 5, *B*–*D*. Our data reveal that AMF does not significantly impact recovery from fast inactivation (Fig. 5*B*). However, for channels in deeper inactivated states (500–5000 ms), AMF slows recovery in a concentration-dependent manner (Fig. 5, *C*–*D*). Notably, this effect is more pronounced at lower AMF concentrations after 5000 ms compared to 500 ms, indicating that AMF has a stronger effect as channels progress into deeper and slower inactivated states.

## Discussion

### Pharmacological targeting of Nav channels

Nav channels are critical components of the electrical signaling system, and their essential role in generating action potentials was recognized as early as the 1950s (41, 42). Consequently, considerable effort has been dedicated to developing/discovering compounds that modulate Nav channel activity (13, 15, 43). As Nav channels transition through various gating conformations, certain regions within the channels undergo substantial changes, while other regions remain relatively structurally rigid (5, 6, 44, 45). Compounds that interact with the regions undergoing significant conformational changes are known as state-dependent modulators (4).

There are several general classes of molecules that inhibit Nav channels. The first class binds to the extracellular part of the PD, a structurally rigid segment of the channel that remains stable across various gating states. Molecules binding here are considered state-independent blockers, with tetrodotoxin being a prominent example (4, 46). The second class consists of compounds that bind to VSD-IV (47, 48, 49, 50). These molecules are highly selective for specific Nav subtypes, a feature attributed to structural differences among VSD-IV segments across different Nav channel subtypes. These compounds are strongly state-dependent, exhibiting >100s to 1000s-fold state dependence due to substantial movements in their binding-sites throughout channel gating. The third class comprises a novel group of highly selective and unusually state-dependent compounds targeting Nav1.8. These compounds, including LTGO-33 (>600 fold selective for Nav1.8 against Nav1.1–7/9), are proposed to bind to an extracellular pocket on VSD-II, formed by S1, S3, S4, and the S3-S4 linker region. Two closely related compounds, VX-150 and VX-548, were recently characterized in detail and exhibited dramatic relief of inhibition during depolarization steps, a phenomenon known as reverse use-dependence (51, 52). VX-548 (Suzetrigine) recently received FDA-approval for acute pain (53, 54). The fourth class represents tamoxifen, which was recently shown to bind to a new binding site at the intracellular interface of a bacterial sodium channel (NavMs) (55).

Finally, the fifth class includes molecules that bind to the LA site (31). These compounds are generally not structurally-selective among Nav channels since the amino acid sequence of the LA site is mostly conserved across Nav channel orthologs (56, 57). However, they are moderately state-dependent (∼10s-fold) as the intracellular side of the PD, where the LA site is located, is less rigid during gating compared to the extracellular side. Notable examples of these compounds include many anticonvulsant drugs, such as phenytoin (15), and antiarrhythmic drugs, such as flecainide (17). This fifth class represents the largest group of Nav-inhibiting molecules currently used in clinical practice.

Classically, LA-binding inhibitors have been divided into two categories: charged and neutral molecules. Charged molecules, like quaternary ammonium ions (QA), can only block the channels when applied intracellularly and require the channels to be open for entry through the V-gate (19, 20, 21, 22). Once inside, the channel can exist in a drug-bound resting or inactivated state. However, due to their positive charge, these molecules cannot permeate through the lipid membrane and the fenestrations leading to the LA site. In contrast, neutral drugs can access the LA site through both pathways, allowing them to bind to the channel in open, inactivated, and resting states (Fig. 5*E*).

### Proposed mechanism of action for *in vitro* inhibition of Nav currents by AMF

CBD shares several similarities with classic neutral LA-binding compounds like lidocaine (4). Functional and structural studies have demonstrated that one of CBD's binding sites is below the selectivity filter inside the channel, although not exactly at the canonical LA site (18, 23, 24). Molecular dynamics simulations suggest that CBD predominantly accesses the LA site through the fenestrations from the lipid phase, which is congruent with its hydrophobicity. Given its high lipophilicity compared to many classic LA drugs, we previously proposed a blocking scheme for CBD in which it interacts with the channel in the resting and inactivated states, supported by its moderate state-dependence (∼10-fold), slow on-rate kinetics, and lack of effect on open-state inactivation (Fig. 5*E*) (4). In the present study, we found that AMF shares several of these features with CBD, but with some key differences. From a functional perspective, our results suggest that AMF may enhance slow inactivation; however, it could also exhibit slower binding and unbinding kinetics rather than directly enhancing the slow inactivation process.

Compared to CBD, AMF exhibits a slightly higher state-dependence (∼30-fold). Our findings show that AMF barely inhibits Nav current at the holding potential of −110 mV, but its apparent potency increases with the depolarization of the holding-potential. This suggests that AMF has a very weak affinity for its binding site when the channels are fully at rest. However, 30 μM AMF produced ∼25% inhibition of the current at −100 mV (Fig. 1*E*). Given that the availability curve of the vehicle indicates only ∼5 to 10% inactivation of the channels at −100 mV (Fig. 4*B*), this level of inhibition implies that AMF interacts not only with the inactivated state but also weakly with the resting state.

We observed that AMF did not exhibit tonic inhibition of Nav currents during the first pulse of our state-dependence experiments (Fig. 1*D*), which would have indicated compound entry into the LA site *via* the aqueous phase through the V-gate (20). This finding supports the hypothesis that AMF primarily accesses its binding site through the fenestrations, with increased inactivation accumulating through repetitive pulsing (Fig. 1*E*). The inhibitory Hill slope of ∼1 further reinforces that AMF likely exerts its inhibitory effects through a single interaction, or the LA site.

An additional possible mechanism for AMF's inhibition of Nav current could involve altering membrane elasticity or stiffness (58). Previous studies have shown that amphiphilic compounds, like Triton X-100, can alter membrane stiffness, leading to a hyperpolarization of the Nav inactivation curve without affecting the voltage dependence of activation (59). A similar property has also been observed with CBD (CBD had the opposite effect to Triton X-100) (18). Given AMF's structure, hydrophobicity, and its biophysical effects on Nav channels described here, our results suggest that upon AMF application *in vitro*, it may 1) alter membrane stiffness, allosterically stabilizing Nav inactivation. As the channels become more inactivated, 2) AMF could then travel through the membrane *via* the fenestrations into the LA site, further directly inhibiting the channels. The molecular details of these pathways warrant investigation in future studies.

### Clinical perspective

Our goal was to determine whether a compound not previously known as a Nav channel inhibitor could inhibit Nav channels *in vitro* using our established criteria. AMF, a phenyl-propyl morpholine derivative (28, 60), was chosen for this purpose. Like many antifungal agents, AMF interferes with ergosterol biosynthesis, with its broad-spectrum fungicidal activity dependent on drug concentration and contact duration (60). While it is not presently possible, based on our *in vitro* study, to propose that inhibition of sodium currents or other similar ionic currents contributes to AMF's antifungal efficacy, it is worth noting that several ion channel subtypes are expressed in fungi (61), making this an area for future research. Given the biophysical profile of AMF described here, it is conceivable that AMF could modulate other Nav channels non-selectively and affect other ion channels at comparable potencies, *in vitro*. Indeed, a recent study suggested that AMF modulates the mitochondrial calcium uniporter (62).

Although AMF is not a highly potent Nav current inhibitor *in vitro*, particularly in comparison to newer classes of VSD-binders that can target these channels at low nanomolar concentrations, AMF's inactivated-state IC_50_ is ∼7 μM, which is comparable to many compounds that target the LA site, including cannabinoids (*e.g.*, CBD and CBG [likely]: ∼1 to 5 μM) (38) and more traditional LAs (*e.g.*, tetracaine), which was shown to block Nav1.1 at around 2 μM from the inactivated state (25).

In conclusion, we have identified a drug not previously recognized as a Nav inhibitor using its physicochemical properties as criteria, guided by our previous findings with CBD, and have described its inhibitory effects on Nav channels. In doing so, we discovered another compound that likely fits the blocking scheme for an ultra-hydrophobic neutral drug targeting the Nav channel LA site.

## Experimental procedures

### Cell culture

A suspension cell line derived from Human Embryonic Kidney 293 cells (Expi293 F, ThermoFisher) was used for automated patch-clamp experiments. Cells were stably transfected with human Nav1.7 channels (63). All cells were incubated on an orbital shaker at 37 °C/8% CO_2_.

### Molecular docking

Molecular docking of AMF into the structure of Nav1.7 was examined into the Nav1.7 structure ([PDB: Protein Data Bank] accession number: 7W9K) using Autodock Vina (64). SwissDock model was used to perform docking, using sampling exhaustivity of 64 (65, 66). To dock AMF into Nav1.7, a search volume of 30 Å x 30 Å x 30 Å around the PD of the channel was considered. This volume range enclosed nearly the whole PD. This yielded the best binding poses of AMF ranked by mean energy score. The list of the top three poses is provided in Table 1.

### Automated patch-clamp

Automated patch-clamp recording was used for all HEK293 experiments. Sodium currents were measured in the whole-cell configuration using a Qube-384 (Sophion A/S) automated voltage-clamp system. The intracellular solution contained (in mM): 120 CsF, 10 NaCl, 2 MgCl_2_, 10 HEPES, adjusted to pH7.2 with CsOH. The extracellular recording solution contained (in mM): 145 NaCl, 3 KCl, 1 MgCl_2_, 1.5 CaCl_2_, 10 HEPES, adjusted to pH7.4 with NaOH. Liquid junction potentials calculated to be ∼7 mV were not adjusted for. Currents were low pass filtered at 5 kHz and recorded at 25 kHz sampling frequency. Series resistance compensation was applied at 100% and leak subtraction was enabled. The Qube-384 temperature controller was used to maintain the recording chamber temperature for all experiments at 22 ± 2 ˚C at the recording chamber. Appropriate filters for cell membrane resistance (typically >500 MOhm), Series resistance (<10 MOhm), and Nav current magnitude (>500 pA at a test pulse from a resting HP of −120 mV) were routinely applied to exclude poor quality recordings. Vehicle controls were run on each plate to enable correction for any compound-independent decrease of currents over time. Baselines were established after 20 min in vehicle. Fractional inhibition was measured as current amplitude from baseline to maximal inhibition after 20-min exposure to test compound unless otherwise noted. Normalized mean inhibition data were fit to the Hill-Langmuir equation:

(1) $$Y={\left[C\right]}^{h}/\left({\text{IC}}_{50}^{h}+{\left[C\right]}^{h}\right)$$

to estimate the half maximal inhibitory concentration (IC_50_ value); where Y is the normalized inhibition, C the compound concentration, IC_50_ the concentration of test compound to inhibit the currents 50%, and h the Hill coefficient. Data analysis was performed using Analyzer (Sophion A/S, Copenhagen, Denmark) and Prism (GraphPad Software Inc., La Jolla, CA, USA) software. All voltage-clamp experiments were done using the Qube.

### Compound preparation

AMF was purchased from Cayman Chemicals. Powdered AMF was dissolved in 100% DMSO to create stock. The stock was used to prepare drug solutions in extracellular solutions at various concentrations with no more than 0.5% total DMSO content.

### Activation protocols

To determine the voltage dependence of activation, we measured the peak current amplitude at test pulse potentials ranging from −120 mV to +25 mV in increments of +5 mV for 500 ms. Channel conductance (G) was calculated from peak I_Na_:

(2) $$G_{\text{Na}}=I_{\text{Na}}/\left(V-E_{\text{Na}}\right)$$

where G_Na_ is conductance, I_Na_ is peak sodium current in response to the command potential V, and E_Na_ (measured on IV relationships) is the Nernst equilibrium potential. Calculated values for conductance were fit with the Boltzmann equation:

(3) $$G/G_{\max}=1/\left(1+\exp\left[V_{1/2}-V_{m}\right]/k\right)$$

where G/G_max_ is the normalized conductance amplitude, V_m_ is the command potential, V_1/2_ is the midpoint voltage and k is the slope.

### Steady-state inactivation protocols

The voltage-dependence of fast-inactivation was measured by preconditioning the channels from −120 to +25 mV in increments of 5 mV for 500 ms, followed by a 10 ms test pulse during which the voltage was stepped to −20 mV. Normalized current amplitudes from the test pulse were fit as a function of voltage using the Boltzmann equation:

(4) $$I/I_{\max}=1/\left(1+\exp\left[V_{1/2}-V_{m}\right]/k\right)$$

where I_max_ is the maximum test pulse current amplitude. The steady-state slow inactivation protocols involved step pulses from −120 mV to 20 mV for 1, 3, 5, or 10 s, followed by 100 ms recovery interval at −120 mV, followed by a test pulse to −20 mV.

### State-dependence protocols

To determine state dependence, potency was measured from three different holding potentials (−110, −100, −90, −80 mV). The protocol started with a holding-potential of −110 mV followed by 180 x 20 ms depolarizing pulses to 0 mV at 1 Hz. Then, the holding-potential was depolarized by 10 mV, and the 180-pulse protocol was repeated until −80 mV was reached.

### Recovery from inactivation protocols

Recovery from inactivation was measured by holding the channels at −120 mV, followed by a depolarizing pulse to −20 mV, then the potential was returned to −120 mV. This was followed by a depolarizing 10 ms test pulse to −20 mV to measure availability. Recovery from inactivation was measured after pre-pulse durations of 20 ms, 500 ms, and 5000 ms and fit with a bi-exponential function of the form:

(5) SpanFast=(Y0−Plateau)∗PercentFast∗0.01

(6) SpanSlow=(Y0−Plateau)∗(100−PercentFast)∗0.01

(7) Y=Plateau+SpanFast∗exp(−KFast∗t)+SpanSlow∗exp(−KSlow∗t)

Where t is time in seconds, Y0 is the Y-intercept at t = 0, KFast and KSlow are rate constants in units the reciprocal of t, PercentFast the fraction of the Y signal attributed to the fast-decaying component of the fit.

### Kinetics of inhibition

The kinetics of the AMF block were measured at three potentials. The channels were held at respective holding potentials followed by pulses to −20 mV. The blocked sodium current was normalized to the vehicle and subsequently fit with a single exponential function:

(8) Y=(Y0−Plateau)∗exp(−K∗t)+Plateau

### Data analysis and statistics

Normalization was performed in order to control the variations in sodium channel expression and inward current amplitude and to be able to fit the recorded data with the Boltzmann function (for voltage-dependences) or an exponential/biexponential function (for time courses of inactivation). The Sophion Qube is an automated electrophysiology instrument that is blinded to cell selections and experimentation, and selection is performed in a randomized manner. All subsequent data filtering and analysis is performed in a non-biased manner, in which automated filters are applied to the entire dataset from a given Qube run. Fitting and graphing were done using Prism nine software (Graphpad Software Inc.) (PRISM, RRID:SCR_005375) (GraphPad, RRID:SCR_000306), unless otherwise noted. All statistical *p*-values report the results obtained from tests that compared experimental conditions to the control conditions. One-way analysis of variance (ANOVA): when multiple concentrations were each being compared to vehicle; or *t* test: when overall two conditions were being compared. A level of significance α = 0.05 was used with *p*-values less than 0.05 being considered to be statistically significant. All values are reported as means ± standard error of means (SEM), standard deviation (SD), or errors in fit, when appropriate, for n recordings/samples. Values are presented as mean ± SEM in the figures for visual clarity, which is common practice in electrophysiology, except for distributive scatter plots which are presented as mean ± SD, with probability levels less than 0.05 considered significant. However, mean ± SD and exact n values are provided in the supplemental data for figures, as required by journal guidelines. The declared group size is the number of independent values, and that statistical analysis was done using these independent values.

## Data availability

All data presented and discussed here are contained within the manuscript.

## Supporting information

This article contains supporting information.

## Conflict of interest

The authors declare that they have no conflicts of interest with the contents of this article.
